# A Multidisciplinary Approach to Evaluate the Effects of Contaminants of Emerging Concern on Natural Freshwater and Brackish Water Phytoplankton Communities

**DOI:** 10.3390/biology10101039

**Published:** 2021-10-13

**Authors:** Paolo Pastorino, Andrea Broccoli, Elisa Bagolin, Serena Anselmi, Andrea Cavallo, Marino Prearo, Monia Renzi

**Affiliations:** 1 Istituto Zooprofilattico Sperimentale del Piemonte, Liguria e Valle d’Aosta, Via Bologna 148, 10154 Torino, Italy; marino.prearo@izsto.it; 2 Bioscience Research Center, Via Aurelia Vecchia, 32, 58015 Orbetello, Italy; andrea.broccoli@bsrc.it (A.B.); serena.anselmi@bsrc.it (S.A.); 3 Dipartimento di Scienze della Vita, Università degli Studi di Trieste, Via Licio Giorgieri 10, 34127 Trieste, Italy; elibagolin@gmail.com (E.B.); mrenzi@units.it (M.R.); 4 CERTEMA Scarl, Strada Provinciale del Cipressino km 10, 58044 Cinigiano, Italy; andrea.cavallo@tostisrl.it

**Keywords:** Bacillariophyceae, Dinoficee, evenness, Shannon–Wiener, nanoparticles, ZnO

## Abstract

**Simple Summary:**

Zinc oxide nanoparticles (ZnO NPs) and potassium dichromate (K_2_Cr_2_O_7_) are two contaminants of emerging concern (CECs; chemicals not commonly monitored in the environment but has the potential to enter the environment and cause known or suspected adverse ecological and/or human health effects). On this path, phytoplankton species, because of their photosynthetic activity, are vital for providing oxygen, and environmental impacts on such organisms can disrupt an entire ecosystem’s integrity. Generally, ecotoxicological assays on monospecific phytoplankton cultures provide useful information about the cellular effects of toxic compounds; however, they have limited application for detecting the effects of environmental pollutants on multiple species communities like in nature. For this reason, in this study, we took for the first time an ecotoxicological (growth rate and inhibition growth rate), ecological (taxonomic identification of species and diversity in communities), and biochemical (photosynthetic pigments) approach to evaluate the effects of ZnO NPs and K_2_Cr_2_O_7_ on natural freshwater and brackish water phytoplankton communities. Results show that both chemicals have negative effects on natural phytoplankton communities with an alteration of the growth rate, species composition, and photosynthetic activity. However, the exposure to ZnO 10 mg/L acts as a growth stimulant for phytoplanktonic communities. Our findings provide evidence for alterations in natural phytoplankton after exposure to such CECs.

**Abstract:**

Ecotoxicological assays on monospecific phytoplankton have limited application for detecting the effects of environmental pollutants on multiple species communities. With this study, we took an ecotoxicological, ecological, and biochemical approach to evaluate the effects of two contaminants of emerging concern (zinc oxide nanoparticles, ZnO NPs, and potassium dichromate, K_2_Cr_2_O_7_) at different concentrations (K_2_Cr_2_O_7_ 5.6–18–50 mg/L; ZnO NPs 10–100–300 mg/L) on natural freshwater and brackish water phytoplankton communities. Cell density and absorbance values decreased in freshwater and brackish water phytoplankton communities after exposure to ZnO NPs (100 mg/L and 300 mg/L only for freshwater), whereas growth rate was increased in both freshwater and brackish water phytoplankton communities after exposure to ZnO NPs 10 mg/L. Differently, there was no clear relationship between concentration and inhibition growth after exposure to K_2_Cr_2_O_7_: the lowest cell density was recorded after exposure to 18 mg/L. Moreover, the evenness index value was lower compared to the other concentrations, indicating the growth of a few, albeit resistant species to higher K_2_Cr_2_O_7_ concentrations. Generally, Bacillariophyceae and Dinoficee were prevalent in phytoplankton cultures after exposure to ZnO NPs and K_2_Cr_2_O_7_. The Shannon-Wiener index was slightly higher in the negative than the positive controls, but diversity was low after all treatments in both ecotoxicological assays. The evenness index was always very close to zero, indicating the numerical predominance of one or very few species. Finally, the decrease in chlorophyll-*a* and pheophytin-*a* in both ecotoxicological assays indicated a change in photosynthetic activity. Our findings provide evidence for alterations in natural phytoplankton after exposure to emerging contaminants that can disrupt an entire ecosystem’s integrity.

## 1. Introduction

Emerging contaminants are strictly defined as “*any synthetic or naturally occurring chemical or any microorganism that is not commonly monitored in the environment but has the potential to enter the environment and cause known or suspected adverse ecological and/or human health effects*” [1]. The majority of emerging contaminants are not totally new or pollutants that have just gained entry into the environment [2,3,4]; most are well-established pollutants with a newly demonstrated toxic effect or mode of action [5]. Hence, the word “emerging” refers not only to the contaminant itself but also to an emerging concern about the contaminant [6]. As such, emerging contaminants are often referred to as chemicals of emerging concern or contaminants of emerging concern (CECs) [7].

Zinc oxide nanoparticles (ZnO NPs) are an example of CECs [8]. Metal-based engineered nanoparticles (MENPs), especially zinc, are widely used in the production of cosmetics, creams, industrial dyes, antibacterial agents, and agrochemicals [9,10]. They are released into the environment from production plants, landfills, or wastewater treatment plants [11]. The fate and spread of MENPs into the environment depend chiefly on their physicochemical characteristics [12]. MENPs can undergo various transformation processes (e.g., aggregation and/or agglomeration, surface absorption, dissolution) that reflect their effects and fate on the biotic compartment [12]. The toxic effects of MENPs on individual organisms are primarily attributable to the formation of reactive oxygen species (ROS) that damage the cell membrane [13].

Chromium (Cr), a transition element ubiquitous in the environment, is commonly found in the form of trivalent chromium Cr (III) and hexavalent Cr (VI) [14]. While the trivalent, insoluble form has relatively low toxicity and is an essential nutrient for organisms, hexavalent chromium, resulting from numerous industrial processes and anthropogenic activities, is highly toxic [15]. Being soluble, it leaches from the soil to the groundwater or to surface waters in high concentrations [16,17]. Cr (VI) in the form of various chemical compounds (i.e., sodium chromate, sodium dichromate, potassium dichromate, calcium chromate, ammonium dichromate, zinc chromate, lead chromate, copper dichromate, magnesium chromate, chromate of mercury, etc.) is used in many industrial processes (i.e., galvanic chrome plating, inks containing chromium-based pigments, pesticides) [18]. Potassium dichromate (K_2_Cr_2_O_7_), for example, is a CEC used as a reagent in industrial (leather dyeing, coloring, tanning) and laboratory applications [19]. It is also used to evaluate the toxicity of toxic substances on model organisms (i.e., *Daphnia magna*) as reported in the literature [20,21].

Phytoplankton are a group of species widely used as model organisms in standardized test methodologies recommended by international regulatory agencies [22,23]. Under current European legislation, data from algal toxicity tests inform the definition of water quality criteria (marine and freshwater) [24]. Phytoplankton species, because of their photosynthetic activity, are vital for providing oxygen for the survival of aquatic and terrestrial species and they occupy a key node in aquatic food webs [25]. One of the most interesting peculiarities of these organisms is their pigments [26]. Chlorophyll (*a*, *b*, *c*) content is widely used as an indicator of physiological stress because photosynthesis is decreased at elevated concentrations of certain pollutants [27]. In particular, chlorophyll-*a* (Chl-*a*) is present in all phytoplankton species, while other accessory pigments are distributed differently across the various classes. Chlorophyll-*a* and pheophytin-*a* (Phe-*a*) are widely applied as marker pigments for total phytoplankton and for general Chl-*a* derivatives, respectively [28].

Because algal ecotoxicological assays in environmental assessments are monospecific, harmful effects are evaluated on individuals of the same phytoplankton species. This type of evaluation is a reductionist approach, however, because complex biological interactions occur between multiple species in the natural environment, where biological communities are affected by chemical pollution. With this study, our objective was to evaluate for the first time the effect of two CECs (ZnO NPs and K_2_Cr_2_O_7_) on two natural phytoplankton communities (brackish water and freshwater). To do this, we took a multidisciplinary approach to ecotoxicological (growth rate and inhibition growth rate), ecological (taxonomic identification of species, calculation of the Shannon–Wiener and the evenness indexes), and biochemical (chlorophyll-*a* and pheophytin-*a* content) assessment.

## 2. Materials and Methods

### 2.1. Experimental Design

Ecotoxicological effects were measured after exposure of two natural phytoplankton cultures (brackish water and freshwater) to ZnO NPs and K_2_Cr_2_O_7_. The purpose of this study was to determine changes in the phytoplankton communities (multiple species) by observing the effects in a combined approach: (a) ecotoxicological-growth rate and growth inhibition test (72 h exposure); (b) ecological-taxonomical identification of species, abundance in phytoplankton classes, and calculation of the Shannon–Wiener and the evenness indexes; (c) biochemical quantification of Chl-*a* and Phe-*a*, indicative of phytoplankton biomass.

The ecotoxicological assays were set up and performed in the laboratory under controlled conditions. Two algal subcultures (freshwater and brackish water) were prepared; after 72 h of exposure subcultures were observed to evaluate the changes in ecotoxicological, ecological, and biochemical parameters; results were compared to natural algal cultures at the same time.

### 2.2. Supply of Phytoplankton Cultures

Samples of the two phytoplankton communities were obtained from two eutrophic environments: a lentic freshwater ecosystem (Osa river, Fonteblanda, Orbetello, Tuscany, Italy) and a brackish lagoon (Orbetello, Tuscany, Italy). The geographical coordinates (latitude and longitude) are 42.5525646° 11.184841° and 42.446880° 11.230121° for Osa river and Orbetello lagoon, respectively. Water samples were collected in October 2020 by immersing a sterile glass bottle (2 L) in water. These environments were selected because located near the laboratory (BsRC Research Centre, Orbetello, Italy) where the ecotoxicological assays were performed. The samples were collected and brought to the laboratory within a few minutes and processed immediately, thus averting physicochemical and biological changes. The physicochemical characteristics of the water environments were salinity 30‰, pH 7.53, dissolved oxygen-O_2_ 7.6 mg/L (brackish water) and pH 7.51, conductivity 206 μS/cm, O_2_ 6.2 mg/L (freshwater).

### 2.3. Ecotoxicological Tests

The ecotoxicological tests were performed following standardized methods for freshwater (ISO 8692:1989) and marine (ISO 10253:2016) unicellular algal growth inhibition test [29,30]. For this study, we used powdered ZnO NPs (CAS n. 1314-13-2, Caelo, particle size <100 nm, surface area 15–25 m^2^/g) and a potassium dichromate solution (0.25 N, Titripur^®^, Sigma-Aldrich, St. Louis, MO, USA).

**Figure 1:**
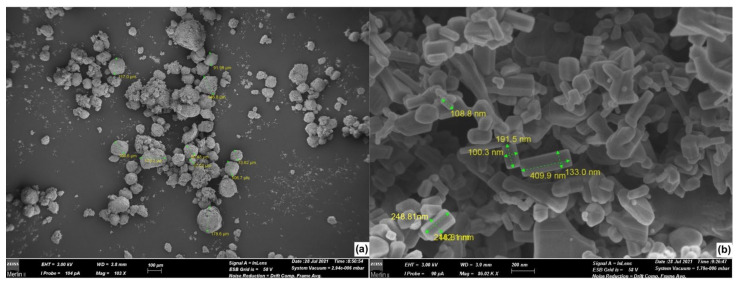
(**a**) Micrometric clusters of ZnO nanoparticles present in untreated dust and (**b**) ZnO nanoparticle size after sonication treatment (FESEM microscopy).

The stock suspension of 500 mg/L ZnO NPs was prepared with Millipore water and treated by sonication with a power of 40 Hz for 20 min to disaggregate micrometric clusters that were present in untreated dust (Figure 1a). The suspension was placed in an ultrasound water bath for 30 min before being diluted to different exposure concentrations [31]. After sonication treatment, an aliquot of the ZnO NPs suspension, at a concentration of 300 mg/L, 100 mg/L, and 10 mg/L (exposure concentrations), respectively, was put in a disposable polystyrene cuvette to determine particle size with FESEM microscopy (Zeiss, mod. Merlin II+WD/ED combined microanalyzer; Figure 1b). Nanoparticles measured (n = 20) showed cubic and parallelepiped shapes with a mean dimension of 58.5 ± 18.4 nm × 77.7 ± 27.0 nm (cubic) and 208.1 ± 143.3 nm × 328.8 ± 132.2 nm (parallelepiped).

The tests (both for brackish and freshwater phytoplankton cultures) entailed preparation of negative controls (pure cultures with the addition of culture medium; *n* = 3), positive controls with potassium dichromate solution (*n* = 3 concentrations; n = 3 replicates) and zinc oxide nanoparticle solution (*n* = 3 concentrations; *n* = 3 replicates). Negative controls for algal cultures were prepared following ISO protocols [29,30]. Briefly, Bold’s basal medium with vitamins and triple nitrate (3NBBM+V) for freshwater [29] and autoclaved filtrate seawater (FSW) plus nutrients as indicated in ISO 10253:2016 for brackish water [30].

Three different concentrations of both toxic compounds were prepared for the positive controls. The K_2_Cr_2_O_7_ concentrations were the same for the freshwater and the brackish water algal test: 50 mg/L, 18 mg/L, and 5.6 mg/L. The ZnO NPs concentrations were: 300 mg/L, 100 mg/L, and 10 mg/L (sonicated as previously described before exposure to tested species). These concentrations were chosen according to the model species (*Pheodactylum tricornutum* for marine and *Pseudokirchneriella subcapitata* for freshwater) for the ISO standard [29,30]. For example, *Pheodactylum tricornutum* has an EC_50_ of 20.53 mg/L K_2_Cr_2_O_7_, whereas the EC_50_ is 1 mg/L for *Pseudokirchneriella subcapitata*. The toxic concentrations were chosen to cause both 100% inhibition of growth and the absence of growth inhibition. Positive and negative controls were placed in 25 mL cuvettes to which the algal inoculums were then added.

### 2.4. Preparation of the Algal Inoculums

For the preparation of the algal inoculums, the water samples were first filtered and then centrifuged to obtain a concentration of approximately 100,000 cells/mL for freshwater [29] and 200,000 cells/mL for brackish water [30] cultures. Briefly, the samples were filtered (cellulose nitrate filter; mesh 0.45 μm) using a vacuum pump. The filter content was then gently removed and washed in a 250 mL beaker; approximately 100 mL of suspension was obtained from the brackish and the freshwater samples. Both inoculums were sieved (mesh 0.063 μm) to retain impurities in the suspension (plant debris, particulate substances, etc.) and prevent feeding by zooplankton. We then inspected under an optical microscope the cells present in the culture and found that all were less than 0.063 μm in diameter; no phytoplankton species were lost. The suspensions were then centrifuged at 22 °C and 10,000 rpm (without loss of cell vitality). A Thoma’s counting chamber was used to measure cell concentration. The cuvettes containing the positive and the negative controls were then filled with the algal suspensions. The algal inoculums (freshwater and brackish water) were also used for taxonomical identification of phytoplankton species and cell count (Section 2.6).

### 2.5. Ecotoxicological Assays

Cuvettes (25 mL) were incubated for 72 h at 18 °C under continuous light (10 klux). The contents were stirred every 24 h to keep the cells in free suspension as much as possible and prevent the transfer of carbon dioxide (CO_2_) from air to water. Cell density was measured by spectrophotometry (Onda UV-30 scan spectrophotometer, optical length 10 cm, Bormac s.r.l., Carpi, Italy) at 670 nm, which corresponds to the maximal absorption for Chl-*a* [32], to determine whether a relationship exists between cell density and absorbance.

### 2.6. Taxonomical Identification of Phytoplankton, Cell Count, and Ecotoxicological Parameters

Phytoplankton were prepared and classified with the Uthermöl procedure [33]. The algal subcultures of the positive and the negative controls were transferred after 72 h incubation into 50 mL Falcon tubes, taking care to resuspend them completely before placing them into the tubes. A drop of Lugol’s solution was added to fix and preserve the algal cells. The tubes were placed in a rack (dark condition) and the content was allowed to settle for 24 h [34]. The algal cells were identified and counted using an Uthermöl sedimentation chamber (Utermöhl Pack, Aquatic BioTechnology, Spain) and an inverted optical microscope (INV100TL, Eurotek, Orma, Italy) at increasing magnification (×20, ×40).

Taxonomic classification to species or family level was performed using taxonomic keys [35,36]. Growth rate and inhibition growth rate were calculated. The specific growth rate (μ) was determined as follows [37,38]:

μ = lnX_L_ − lnX_0_/t_L_ − t_0_ (1)

where X_L_ is the number of cells at time t_L_ (3 days, 72 h), and X_0_ the initial number of cells at time t_0_.

The growth inhibition rate (I%) was calculated according to OECD 2011 guidelines [37]:

Inhibition (%) = [(μC − μT)/μC] ×100(2)

where μC is the mean for average specific growth rate (μ) in the controls and μT is the average specific growth rate for the treatment replicate.

Finally, the Shannon–Wiener (H′) and the evenness (E) indexes were calculated using the equations reported in the literature [39]. These indexes were selected since they are considered suitable for analyzing phytoplankton community diversity [40].

### 2.7. Biochemical Analyses

Chl-*a* and Phe-*a* levels in cell cultures were determined following the APAT CNR IRSA 9020 [41] method at the end of the inhibition of growth test (72 h). The negative and the positive controls were filtered on glass fiber filters (pore diameter, 0.70 μm) by vacuum pump, extracted with acetone 90% (Sigma-Aldrich, analytic grade) kept on ice and in the dark for 2 h, at 4 °C to prevent pigment alterations. The samples were then centrifuged for 3 min at 22 °C, 10,000 rpm. The supernatant was collected, and spectrophotometric absorbance measured at 630, 647, 664, 750 nm (Onda UV-20 UV/Vis spectrophotometer, Bormac). To extract Phe-*a*, 90 μL of HCl (0.1 M) were added; after 5 min of acid exposure, readings were performed at 665 and 750 nm. The content was calculated according to the APAT CNR IRSA 9020 [41] method as follows:

Chl-*a* = {[11.85 (Abs 664–750) − 1.54 (Abs 647–750) − 0.08 (Abs 630–750)] v}/V·L

Phe-*a* = {26.7 [1.7 (Abs 665a–750a) − (Abs 664–750)] v}/V·L

where Abs is the absorbance measured at the set wavelength, v is the volume (mL) of acetone extract, V is the sample volume (L), L is the optical path (cm), “*a*” denotes acidified. Data are expressed as μg/L per compound. The limit of quantification (LOQ) was 0.001 μg/L.

### 2.8. Quality Assurance and Quality Control (QA/QC)

A QA/QC protocol was applied during the experiments to ensure the methodological quality of the experimental results. The Bioscience Research Centre is a certified laboratory (ISO 9001:2015) and applies strict control procedures under UNI EN ISO 17025:2018 guidelines to ensure data quality. The QA/QC tests were performed as described in the reference methods (ISO 8692:1989 and ISO 10253:2016) [29,30]. Tests were performed following the general quality criteria applied by the laboratory including positive and negative controls during experiments and a metrologically traceable approach based on the use of LAT calibrated instrumentation.

### 2.9. Statistical Analysis

The Kolmogorov–Smirnov test was performed to determine whether our dataset was well-modeled by normal distribution. Since the null hypothesis for normal distribution could not be rejected, the non-parametric Kruskal–Wallis was used to compare cell density, absorbance, the Shannon–Wiener, and the evenness index, Chl-*a*, Phe-*a*, and growth rate between the negative and the positive controls (K_2_Cr_2_O_7_ and ZnO treatment) followed by the Conover–Iman post hoc test. The Mann–Whitney U test was used for pairwise comparisons when values were <LOQ. Simple linear regression was used to check the strength of the correlation (R^2^) between absorbance (670 nm) (dependent variable) and cell density (independent variable). Principal component analysis (PCA) was performed to check for trends in phytoplankton species abundance (number of cells) between negative controls (untreated cultures) and exposed cultures (K_2_Cr_2_O_7_ and ZnO NPs treatment). PCA plots were also created to illustrate the clustering of phytoplanktonic species abundance in the negative controls and the exposed cultures. The criterion for significance was set at *p* < 0.05. Statistical analyses were performed using R software (version 1.1.463, RStudio, Inc., Boston, MA, USA).

## 3. Results

### 3.1. Phytoplankton Community in the Inoculum

#### 3.1.1. Freshwater

**Figure 2:**
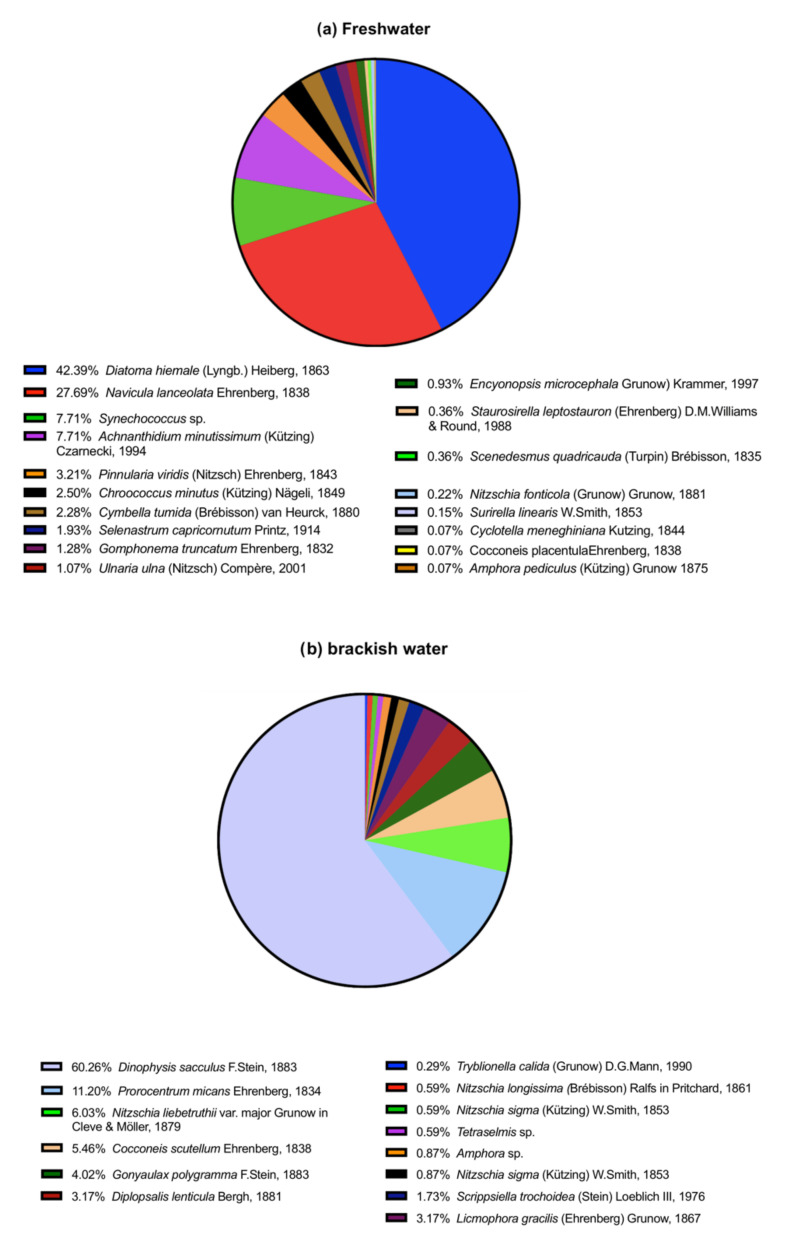
Phytoplankton species (in percentage; %) in freshwater (**a**) and in brackish water (**b**) inoculums.

The freshwater inoculum contained 18 species in the class Bacillariophyceae (87.5%), Cyanophyceae (10.2%), and Chlorophyceae (2.3%) (Figure 2a). The Shannon–Wiener and the evenness indexes values were 1.79 and 0.10, respectively.

#### 3.1.2. Brackish Water

The brackish water inoculum contained 15 species in the class Dinophyceae (81.6%), Bacillariophyceae (17.8%), and Prasinophyceae (0.6%) (Figure 2b). The Shannon–Wiener and the evenness indexes values were 1.54 and 0.10, respectively.

### 3.2. Freshwater–K_2_Cr_2_O_7_ and ZnO NPs Ecotoxicity Test

There was a significant difference in cell density (cell/mL) between the negative and the positive controls (Kruskal–Wallis test; *p* = 0.001), with a significantly lower cell density after Cr 18 treatment compared to the negative control (Conover–Iman test; *p* = 0.003) (Table 1). There was a significant difference in absorbance (670 nm) between the negative and the positive controls (Kruskal–Wallis test; *p* = 0.001), with lower measurement after Cr 18 treatment compared to the negative control (Conover–Iman test; *p* = 0.01). Linear regression analysis showed a significant linear relationship between cell density and absorbance (R^2^ = 0.99; y = 7.662e^−005^× −0.07289; *p* < 0.001). The number of phytoplankton species ranged from 12 (after exposure to Cr 5.6 and Cr 18) to 13 (after exposure to Cr 50) and 14 (negative control). There was no significant difference in the Shannon–Wiener or the evenness indexes between the negative and the positive controls (Kruskal–Wallis test; *p* > 0.05). There was no significant difference in Chl-*a*, pheophytin, and growth rate between the negative and the positive controls (Kruskal–Wallis test; *p* > 0.05). Finally, growth inhibition was 14.4%, 11.7%, and 7.3% after exposure to Cr 18, Cr 5.6, and Cr 50 treatment, respectively.

There was a significant difference in cell density (cell/mL) between the negative and the positive controls (Kruskal–Wallis test; *p* = 0.0001) and a significant difference between the negative control and exposure to Zn 10 (Conover–Iman test; *p* = 0.001) and Zn 300 (Conover–Iman test; *p* = 0.008) (Table 1). Cell density (4945 cell/mL) was higher after Zn 10 treatment. There was a significant difference in absorbance between the negative and the positive controls; absorbance was lower (0.027) after Zn 300 treatment and higher (0.305) after Zn 10 treatment. The number of phytoplankton species was 12 after Zn 100 and Zn 300 treatment and 14 in the negative control and after Zn 10 treatment. The Shannon–Wiener and the evenness indexes were similar for the positive and the negative controls; there was no significant difference between the negative control and treatments (Kruskal–Wallis test; *p* < 0.05). There was a significant difference in Chl-*a* between the negative control and treatments (Kruskal–Wallis test; *p* = 0.001); Chl-*a* was higher after Zn 10 treatment (Conover-Iman test; *p* = 0.007). There was no difference in pheophytin between the negative controls and treatments (Kruskal–Wallis test; *p* > 0.05). There was a significant difference in growth rate between the negative control and treatments (Kruskal–Wallis test; *p* = 0.002), with a significant difference between the negative control and after Zn 10 and Zn 300 treatment (Conover–Iman test; *p* = 0.004 for both). Finally, inhibition growth was 55.03% and 11.79% for Zn 300 and Zn 100, respectively, and −143.81 after Zn 10 treatment.

### 3.3. Trend in Ecotoxicological Indexes: Freshwater

**Figure 3:**
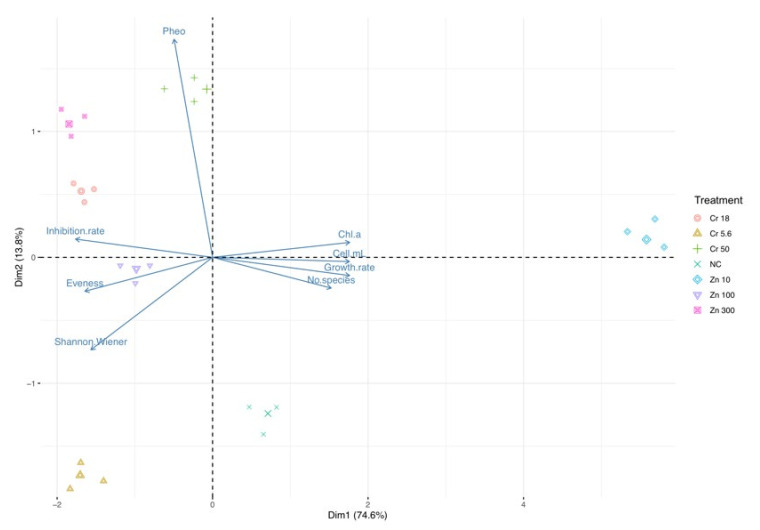
Principal component analysis. Trend in ecotoxicological indexes (pheophytin-Pheo, chlorophyll-*a*-Chl-*a*, inhibition rate, evenness, Shannon–Wiener, inhibition rate, growth rate, number of species, cell density-cell/mL) across treatments (zinc oxide nanoparticles-Zn and potassium dichromate-Cr) in freshwater phytoplankton community (largest symbol = average value).

Principal component analysis disclosed similar trends in the ecotoxicological indexes for Cr_2_O_7_ and ZnO treatments. The first PCA (Figure 3) showed that the first (Dim1) and the second (Dim2) components accounted for meaningful amounts of the total variance (90.4%). Dim1 explained 74.6% of the total variance and Dim2 13.8%. Chl-*a*, cell density (cell/mL), growth rate, and the number of species correlated with Dim 1, whereas inhibition rate and the evenness index were negatively correlated with Dim1. Pheophytin-*a* (Pheo) was positively correlated with Dim2. Zn 10 treatment is located in the right part of the plot in an area of increasing Chl-*a*, cell density (cell/mL), growth rate, and the number of species, and decreasing inhibition rate and evenness indexes. The other treatments are located in the left part of the biplot in an area of higher inhibition rate.

### 3.4. Brackish Water: K_2_Cr_2_O_7_ and ZnO Ecotoxicity Test

There was a significant difference in cell density (cell/mL) between the negative and the positive controls (Kruskal–Wallis test; *p* = 0.001), with a significantly lower density after Cr 18 treatment compared to the negative control (Conover–Iman test; *p* = 0.01) (Table 2).

There was a significant difference in absorbance (670 nm) between the negative and the positive controls, with a significantly lower measurement after Cr 18 treatment compared to the negative control (Conover–Iman test; *p* = 0.01). Linear regression analysis showed a significant linear relationship between cell density and absorbance (R^2^ = 0.90; y = 0.0001534 *× + 8.756e^−005^; *p* = 0.004). The number of phytoplankton species was 11 in the negative control and after Cr 50 treatment and 12 after Cr 18 and 13 after Cr 5.6 treatment, respectively. There was a significant difference in the Shannon–Wiener and the evenness index between the negative and the positive controls. There was a significant difference in both indexes between the negative control and after CR 50 treatment (Conover–Iman test; *p* = 0.01). Chl-*a* was detected only in the negative control and after Cr 5.6 treatment. Measurements <LOQ (0.001 μg/L) were found after Cr 18 and Cr 50 treatments. Pheophytin was detected only in the negative controls; measurements were <LOQ (0.001 μg/L) after all treatments. There was no significant difference in growth rate between the negative and the positive controls, albeit with a lower rate (0.44) after CR 18 treatment. Finally, inhibition growth was 22.82% after Cr 18 treatment; negative rates were recorded after Cr 50 (−2.27%) and Cr 5.6 (2.39%) treatments.

There was a significant difference in cell density (cell/mL) between the negative and the positive controls (Kruskal–Wallis test; *p* = 0.0001), with a significant difference between the negative control and after Zn 100 (Conover–Iman test; *p* = 0.01) and Zn 10 treatment (Conover–Iman test; *p* = 0.01). Cell density was higher after Zn 10 treatment. All microalgae died after Zn 300 treatment; no results are reported (Table 2). There was a significance difference in absorbance between the negative and the positive controls (Kruskal–Wallis test; *p* = 0.002), with lower measurements (0.023) after Zn 100 treatment and higher (0.249) after Zn 10 treatment. The number of phytoplankton species was 9 after Zn 100 treatment and 11 and 13 in the negative control and after Zn 10 treatment, respectively. The Shannon–Wiener index for the negative control was higher (2.02), but the Kruskal-Wallis test did not disclose a difference (p > 0.05) between the negative control and the two treatments (Zn 10 and Zn 100). The evenness index showed no difference between the negative control and the treatments. The evenness index was higher (0.21) after Zn 100 treatment. There was a significant difference in Chl-a and pheophytin (Mann–Whitney U test; *p* = 0.006) between the negative control and after Zn 10 treatment. There was a significant difference in growth rate between the negative control and treatments (Kruskal–Wallis test; *p* = 0.003), with a significant difference between the negative control and after Zn 100 treatment (Conover–Iman test; *p* = 0.01). Growth inhibition was 54.65% after Zn 100 treatment and −14.49% after Zn 10 treatment.

### 3.5. Trend in Ecotoxicological Indexes: Brackish Water

**Figure 4:**
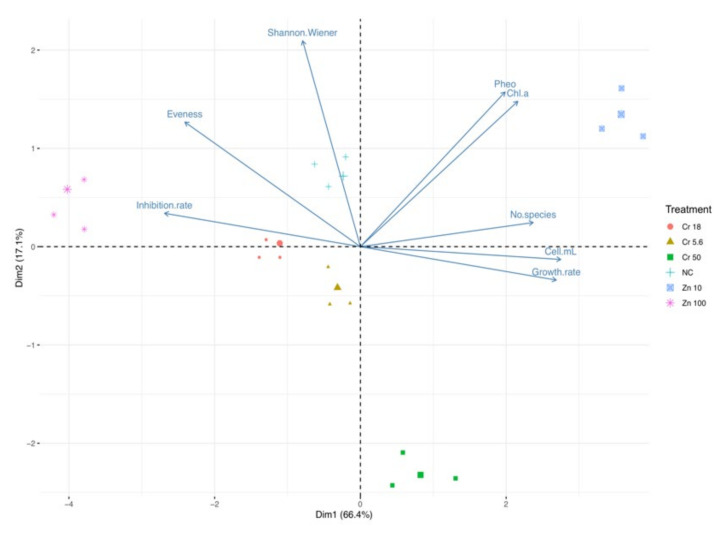
Principal component analysis. Trend in ecotoxicological indexes (pheophytin-Pheo, chlorophyll-*a*–Chl-*a*, inhibition rate, evenness index, Shannon–Wiener index, inhibition rate, growth rate, number of species, cell density–cell/mL across treatments (zinc oxide nanoparticles–Zn and potassium dichromate–Cr) in brackish water phytoplankton community (largest symbol = average value).

Principal component analysis disclosed a trend in ecotoxicological indexes very similar to that recorded for freshwater. The second PCA (Figure 4) showed that the first (Dim1) and the second (Dim2) components accounted for meaningful amounts of the total variance (83.5%). Dim1 explained 66.4% of the total variance and Dim2 17.1%. Chl-*a*, pheophytin, cell density (cell/mL), growth rate, and number of species correlated with Dim1, whereas inhibition rate and the evenness index were negatively correlated with Dim1 and the Shannon–Weiner index was positively corrected with Dim2. Zn 10 treatment is located in the right part of the plot in an area of increasing Chl-*a*, pheophytin, cell density (cell/mL), growth rate, and number of species, and a decrease in inhibition rate and the evenness index. The other treatments are located in the left part of the biplot in an area of increasing inhibition rate and the evenness index.

### 3.6. Trend in Phytoplankton Abundance

#### 3.6.1. Freshwater

**Figure 5:**
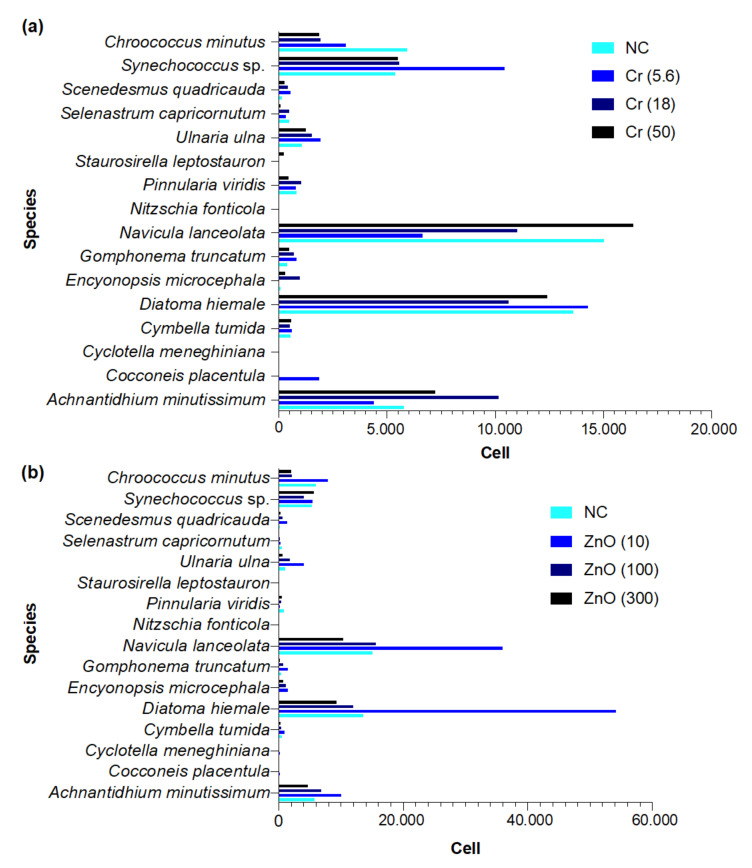
Mean number of cells recorded at each concentration of Cr_2_O_7_ (Cr; (**a**)) and ZnO (Zn; (**b**)), negative control (NC) included in freshwater phytoplankton community.

The mean number of cells recorded at each concentration of K_2_Cr_2_O_7_ and ZnO, including the negative control, is presented in Figure 5a,b. The species belonged to the classes Bacillariophyceae, Chlorophyceae, and Cyanophyceae.

**Figure 6:**
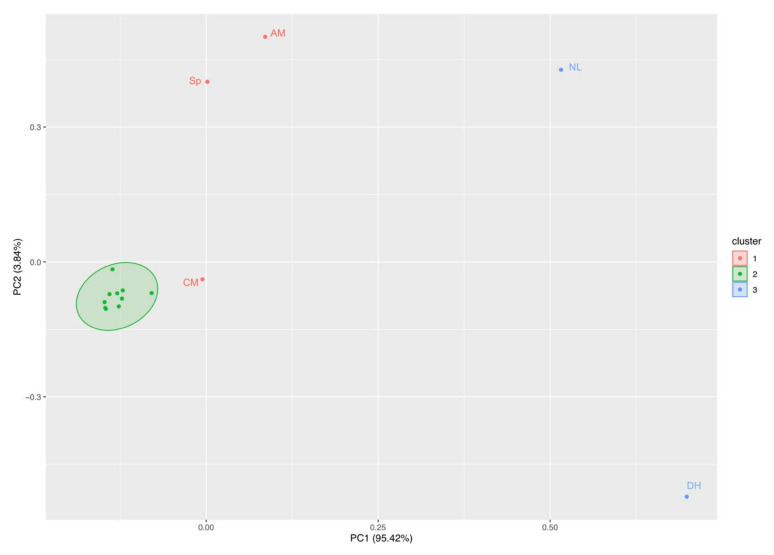
Principal component analysis of freshwater phytoplankton abundance across treatments (Cr_2_O_7_ and ZnO). The main cluster (green) contains the majority of the species that were similar in abundance. The red cluster contains only three species: *Achnantidhium minutissimum*–AM, *Synechococcus* sp.–Sp, and *Chroococcus minutus*–CM. The blue cluster contains *Navicula lanceolata*–NL and *Diatoma hiemale*–DH.

PCA of phytoplankton species abundance yielded three main clusters (Figure 6). The main cluster (green) contains the majority of the species that were similar in abundance across treatments (K_2_Cr_2_O_7_ and ZnO). The red cluster contains only three species: *Achnantidhium minutissimum*, *Synechococcus* sp., and *Chroococcus minutus*. Finally, the blue cluster contains only *Navicula lanceolata*, and *Diatoma hiemale*. These two clusters contain the species with the highest abundance across all treatments.

#### 3.6.2. Brackish Water

**Figure 7:**
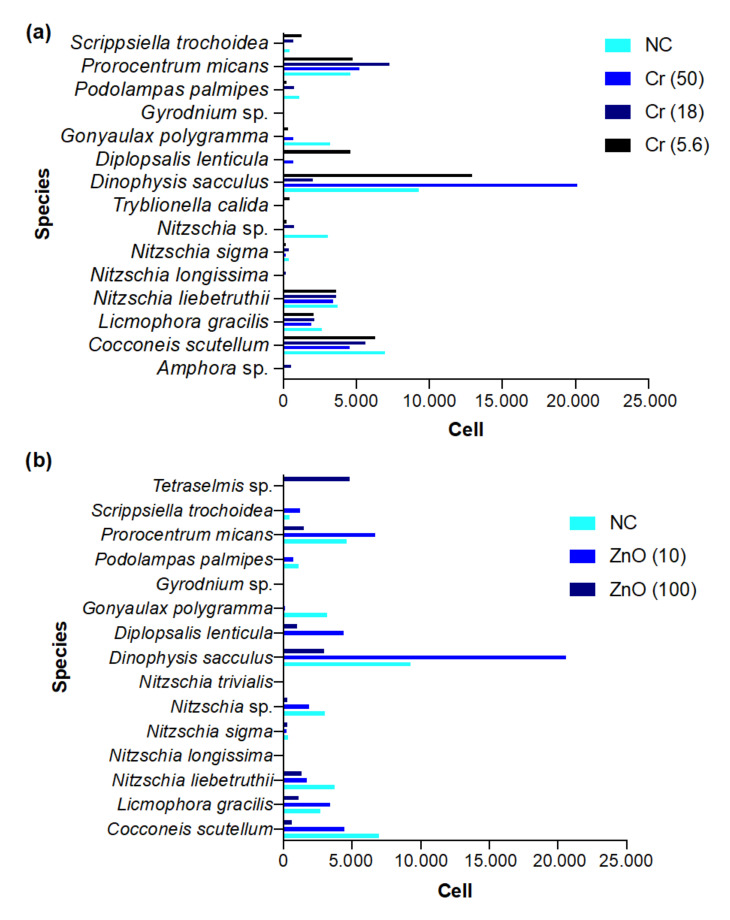
Mean number of cells recorded at each concentration of Cr_2_O_7_ (Cr; (**a**)) and ZnO (Zn; (**b**)), negative control (NC) included, in brackish water phytoplankton community.

The mean number of cells recorded at each concentration of K_2_Cr_2_O_7_ and ZnO, including the negative control, is presented in Figure 7a,b. Species belonged to the classes Bacillariophyceae, Dinophyceae, and Prasinophyceae.

**Figure 8:**
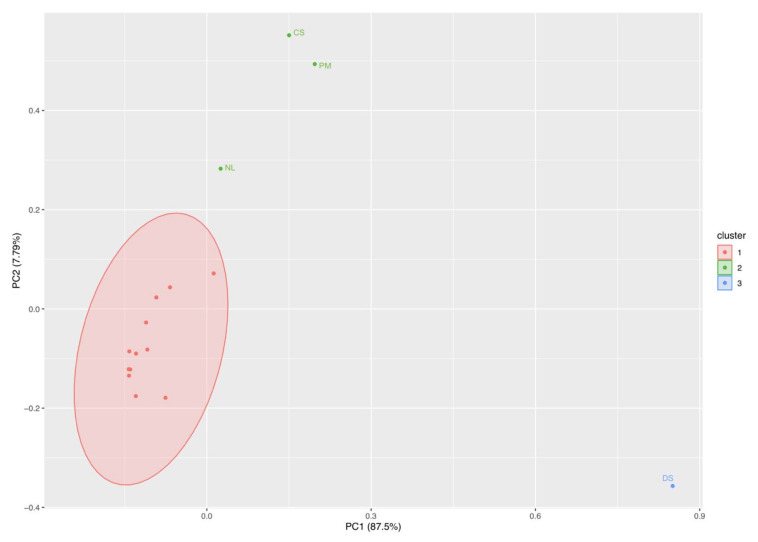
Principal component analysis of brackish water phytoplankton abundance. The main cluster (red) contains the majority of the species that were similar in abundance across treatments (Cr_2_O_7_ and ZnO). The red cluster contains only three species: *Cocconeis scutellum*–CS, *Nitzschia* liebetruthii–NL, and *Prorocentrum micans*–PM. The blue cluster contains *Dinophysis sacculus*–DS.

PCA of phytoplankton species abundance yielded three main clusters (Figure 8). The main cluster (red) contains the majority of the species that were similar in abundance across treatments (K_2_Cr_2_O_7_ and ZnO). The red cluster contains only three species: *Cocconeis scutellum*, *Nitzschia liebetruthii*, and *Prorocentrum micans*. Finally, the blue cluster contains only *Dinophysis sacculus*. These last two clusters contain the species with the highest abundance across all treatments.

## 4. Discussion

### 4.1. Effect of ZnO NPs and K_2_Cr_2_O_7_ on Natural Phytoplankton Communities

The main aim of the present study was to understand the effect of environmentally relevant exposure to waterborne emerging micropollutants (ZnO NPs and K_2_Cr_2_O_7_) on phytoplankton communities. Natural phytoplankton communities were sampled in natural environments. Both the freshwater and the marine inoculum had the highest number of species (n = 18 for freshwater and n = 15 and brackish water, respectively) compared to the negative controls and after ZnO and K_2_Cr_2_O_7_ treatments. The inoculum is the natural condition where a higher number of species can survive in such conditions. The presence of a toxic compound, even at low concentrations, alters the natural conditions in which a small number of species can survive, generally those most resistant to harsh conditions [42]. The negative control also had a low number of species (n = 14 for freshwater and n = 11 for brackish water, respectively) compared to the inoculum, since the environmental conditions reproduced in the laboratory do not permit all species to survive.

In this study, cell count was performed using an Uthermöl sedimentation chamber. Quantification of phytoplankton usually involves time-consuming methods, such as direct cell count under the microscope or measurement of cellular mass [43]. Our findings revealed a significant association between cell density and absorbance, demonstrating that indirect methods that correlate algal density to absorbance at specific wavelengths are not only reliable but also easy to set up for future monitoring systems.

Inhibition growth was observed in freshwater and brackish water phytoplankton communities after exposure to ZnO at 100 mg/L and 300 mg/L (only for brackish water). Previous studies reported the ecotoxic effect of ZnO NPs on various freshwater and marine algal species (monocultures). Miao et al. [44] studied the effects on *Thalassiosira pseudonana* exposed to ZnO NPs and found that dissolution of Zn^2+^ could be the major cause for its toxicity, as revealed in growth inhibition and lower chlorophyll content. Bhuvaneshwar et al. [45] reported that ZnO NPs toxicity was strongly dependent on particle morphology and stability. Aravantinou et al. [38] investigated the effect of ZnO NPs on freshwater (*Chlorococcum* sp. and *Scenedesmus rubescens*) and marine (*Dunaliella tertiolecta* and *Tetraselmis suesica*) microalgae species and found that NPs appeared to have toxic effects on all species tested, depending on species, exposure time, NPs concentration, and mainly the culture medium. Gunawan et al. [46] studied the effect of ZnO particles on the freshwater green alga *Chlamydomonas reinhardtii* even at very low concentrations (8-day EC_50_ ≥ 0.01 mg/L). Our results showed that exposure to ZnO 10 mg/L significantly increased growth rate and absorbance in the freshwater and the brackish water phytoplankton communities, which showed negative growth inhibition rates. This important finding leads us to assume that such ZnO concentrations act as a growth stimulant for phytoplanktonic communities. Kumar et al. [47] found that zinc (15 mg/L) can act as a growth stimulant for *Tetraselmis* sp. and *Chlorella marina* exposed to increasing Zn concentrations (5–1500 mg/L). Zn is a basic component of various enzymes involved in photosynthesis and algae metabolism, including carbon anhydride, acidic phosphatase, and alkaline phosphatase [48]. Wong and Chau [48] found that the growth rate of *Ankistrodesmus falcatus* was greatly reduced after exposure to ZnCl_2_, whereas 5 μg/L of Zn stimulated algal growth.

We found no clear relationship between concentration, growth rate, and inhibition growth rate after exposure to potassium dichromate. This finding is shared by Kusk and Nyholm [49] who assessed the sensitivity of natural marine phytoplankton and five species of marine microalgae to different K_2_Cr_2_O_7_ concentrations. They found much broader growth inhibition rates in natural phytoplankton compared to single species due to the variable sensitivity of different taxa to K_2_Cr_2_O_7_. In a recent study on the ecotoxicological effects of hexavalent chromium on phytoplankton and bacterioplankton from Río de la Plata (South America), some cyanobacteria species (*Anabaenopsis circularis*, *A. incerta*, *Merismopedia convoluta*, *Planktothrix agardhii*) were not negatively affected by exposure to chromium but rather increased their relative abundance between 50 and 320% [22]. In our study, cell density after exposure to 18 mg/L of K_2_Cr_2_O_7_ was significantly higher compared to the other concentrations. Moreover, both the Shannon–Wiener and the evenness index were decreased, indicating the presence of a few but well-adapted species: *Navicula lanceolata* and *Diatoma hiemale* (freshwater species) and *Dinophysis sacculus* (brackish water species) rapidly spread at this concentration, indicating resistance against Cr^6+^ concentration.

High cellular toxicity of Cr^6+^ is expected since it penetrates the cytoplasm and has adverse cellular and genotoxic effects [50]. There are, however, resistance mechanisms that allow algae (i.e., *Scenedesmus acutus*) to cope with chromate toxicity and enable them to survive harsh environmental conditions [51]. Furthermore, some bacteria are able to reduce and detoxify chromium from contaminated environments [52]. Plots obtained from PCA analysis showed that a few algal species were abundant after all treatments, also at high concentrations. Bacillariophyceae was prevalent in the ecotoxicological assay on freshwater phytoplankton, which is widely distributed in aquatic environments [53]. The most abundant species in this class were *Navicula lanceolata*, *Diatoma hiemale*, and *Achnanthidium minutissimum*, typical freshwater diatoms. Cyanophyceae of the genera *Synechococcus* and *Chroococcus* were also found after all treatments, albeit at lower percentages.

Regarding the ecotoxicological assay on brackish phytoplankton, Dinoficee was prevalent with the species *Dinophysis sacculus* (very common along the Mediterranean coast), *Prorocentrum micans* (present in the Mediterranean Sea), and *Scrippsiella trochoidea*, one of the most planktonic scrippsielloid dinoflagellates [54]. Bacillariophyceae was also abundant with the species *Cocconeis scutellu* (typical of both brackish and marine waters) and *Licmophora gracilis*, a cosmopolitan species [37].

Although the Shannon-Wiener index was slightly higher for the negative compared to the positive controls, diversity across treatments was rather low. In addtion, the evenness index was similar for the negative and the positive controls, very close to zero, indicating a condition in which one or very few species were numerically predominant. This hypothesis was confirmed by multivariate analysis (PCA cluster plots) which showed that the species *Achnanthidium minutissimum*, *Diatoma hiemale*, *Navicula lanceolata*, *Synechococcus* sp., *Chroococcus minutus* (freshwater community) and *Dinophysis sacculus*, *Prorocentrum micans*, *Cocconeis scutellum*, *Nitzschia liebetruthii*, and *Prorocentrum micans* (brackish water community) were much more abundant than the other species across all treatments, even at a high concentration of Zn and Cr. A plausible explanation is that certain algae have a high degree of phenotypic or genotypic tolerance to metal toxicity by virtue of their ability to reduce uptake or exclusion from the cell or to implement an efflux mechanism, via biotransformation (conversion of toxic metals into a less toxic form), sequestration, detoxification, prevention of synthesis, ROS production [55]. For example, the algae *Chlorella vulgaris* is able to convert Cr(VI) into the less toxic form Cr(III) [56].

Finally, chlorophyll-*a* and pheophytin-*a* contents were used as an indicator of physiological stress because photosynthesis is known to be decreased at elevated concentrations of certain pollutants [57]. The decrease in both pigments in both ecotoxicological assays indicated a change in photosynthetic activity. There was a significant increase in both phytoplankton communities after exposure to 10 mg/L of ZnO NPs, demonstrating growth promotion and bio-stimulation of the cultures.

### 4.2. Ecological Implications

A pivotal trophic level impacted by pollution in the aquatic environment is phytoplankton, known for their role as microscopic primary producers and base of aquatic food webs [25]. Pollution affects phytoplankton communities at different levels: abundance, growth strategies, dominance, and succession patterns. [58]. Species diversity is a good measure for ecosystem functioning and the stress exerted by environmental pollutants. In this study, we observed how few phytoplankton species were abundant after all ZnO NPs and K_2_Cr_2_O_7_ treatments, even at high concentrations. On this path, it was found how pollution-mediated losses of phytoplankton diversity may have direct detrimental effects on the aquatic primary production [59].

Many studies on algal species and specific pollutants have been published. For example, Baho et al. [58] assessed the ecological effects of pharmaceuticals and personal care products (PPCPs) on natural phytoplankton community structure in environmentally relevant scenarios and found how the two highest treatment levels of PPCPs were associated with decreased abundance of the most dominant size class (nano-phytoplankton: 2–5 μm), leading to a flattening of the size spectra slope. Echeveste et al. [60] reported sets of experiments performed in the Atlantic Ocean and showed how a complex mixture of organic pollutants have an important toxic effect on phytoplankton abundances, viability, and concentrations of chlorophyll-*a*. Other studies have described effects upon population growth or photosynthesis and indicate that, generally, phytoplankton communities are as sensitive to pollutants as animals [61]. Growth and photosynthesis are closely related, each being a function of the utilization of light and nutrients [62]. In this context, contaminants could potentially decrease the efficiency of the carbon transfer mediated by the phytoplankton food web and shift the pathways towards heterotrophic bacteria via the microbial loop [63]. Contaminants can also potentially alter the aquatic food web by shifting the edible portion that is appealing to grazers towards larger non-edible species [64] and can lead to blooms of larger phytoplankton [65]. Moreover, a perturbation of the natural phytoplanktonic communities and occurrence of toxic or potentially harmful algae were observed in polluted sites from Lebanese coastal waters (Eastern Mediterranean Sea) [66], highlighting the influence of wastewater effluents on the seawater equilibrium and thus on marine biodiversity.

## 5. Conclusions

The ecotoxicological effects of ZnO NPs and K_2_Cr_2_O_7_ on natural phytoplankton communities were evaluated here for the first time. Our findings show that both compounds have negative effects on natural phytoplankton communities. While ZnO NPs at 10 mg/L induced a biostimulatory effect on phytoplankton cultures, exposure to K_2_Cr_2_O_7_ at 50 mg/L induced the selection of a few, toxin-resistant species. This study showed that the use of a multidisciplinary approach can provide new insights and stimulate future research on the effects of diffuse chemical pollution at the community level. Thus, our findings can help to improve environmental management and policies to protect the aquatic ecosystem and safeguard ecosystem services in a long-term perspective. Further studies, especially at the molecular level (resistance to toxicants), are needed to better understand the negative effects of emerging contaminants on phytoplankton communities.

## Figures and Tables

**Table 1:** Results of ecotoxicological assay of potassium dichromate-K_2_Cr_2_O_7_ (Cr; a) and zinc oxide nanoparticles-ZnO NPs (Zn; b) on freshwater phytoplankton community. Lowercase letters (^a^, ^b^) denote differences revealed by Conover–Iman post hoc or Mann–Whitney tests. NC denotes negative control. Phe-*a* = pheophytin-*a*; Chl-*a* = chlorophyll-*a*.

Test	NC	Cr 50 mg/L	Cr 18 mg/L	Cr 5.6 mg/L
Total cell	49,288 ± 20.87	47,025 ± 25.89	44,946 ± 36.98	45,723 ± 45.73
Density (cell/mL)	1972 ^a^ ± 16.78	1881 ^a^ ± 17.45	1798 ^b^ ± 89.12	1829 ^a^ ± 19.45
Absorbance	0.079 ^a^ ± 0.004	0.069 ^a^ ± 0.002	0.054 ^b^ ± 0.007	0.064 ^a^ ± 0.009
Number of species	14	13	12	12
Shannon-Wiener	1.99 ^a^ ± 0.78	1.73 ^a^ ± 0.67	1.91 ^a^ ± 0.58	1.94 ^a^ ± 0.29
Evenness	0.16 ^a^ ± 0.09	0.13 ^a^ ± 0.08	0.16 ^a^ ± 0.12	0.16 ^a^ ± 0.09
Chl-*a* (μg/L)	0.002 ^a^ ± 0.001	0.002 ^a^ ± 0.002	0.002 ^a^ ± 0.001	0.001 ^a^ ± 0.001
Phe-*a* (μg/L)	0.003 ^a^ ± 0.002	0.001 ^a^ ± 0.002	0.001 ^a^ ± 0.002	0.003 ^a^ ± 0.001
Growth rate	0.21 ^a^ ± 0.08	0.20 ^a^ ± 0.09	0.18 ^a^ ± 0.08	0.19 ^a^ ± 0.08
Growth inhibition (%)	-	7.3 ± 2.23	14.4 ± 3.34	11.7 ± 2.16
**Test**	**NC**	**Zn 300 mg/L**	**Zn 100 mg/L**	**Zn 10 mg/L**
Total cell	49288 ± 20.87	34665 ± 78.67	45707 ± 18.43	123634 ± 21.35
Cell density (cell/mL)	1972 ^a^ ± 16.78	1387 ^b^ ± 28.54	1828 ^a^ ± 15.67	4945 ^b^ ± 12.71
Absorbance	0.079 ^a^ ± 0.020	0.027 ^b^ ± 0.002	0.090 ^a^ ± 0.001	0.305 ^b^ ± 0.04
Number of species	14	12	12	14
Shannon–Wiener	1.89 ^a^ ± 0.78	1.77 ^a^ ± 0.67	1.80 ^a^ ± 0.56	1.58 ^a^ ± 0.23
Evenness	0.13 ^a^ ± 0.09	0.15 ^a^ ± 0.09	0.15 ^a^ ± 0.08	0.11 ^a^ ± 0.06
Chl-*a* (μg/L)	0.002 ^a^ ± 0.001	0.001 ^a^ ± 0.001	0.002 ^a^ ± 0.001	0.008 ^b^ ± 0.002
Phe-*a* (μg/L)	0.003 ^a^ ± 0.002	0.003 ^a^ ± 0.002	0.002 ^a^ ± 0.001	0.001 ^a^ ± 0.001
Growth rate	0.21 ^a^ ± 0.08	0.10 ^b^ ± 0.09	0.19 ^a^ ± 0.13	0.52 ^b^ ± 0.18
Growth inhibition (%)	-	55.03 ± 6.67	11.79 ± 3.45	−143.81 ± 56.24

**Table 2:** Results of ecotoxicological assay of potassium dichromate-K_2_Cr_2_O_7_ (Cr; a) and zinc oxide nanoparticles-ZnO NPs (Zn; b) on brackish water phytoplankton community. NC denotes negative control. Lowercase letters (^a^, ^b^) denote differences revealed by Conover–Iman post hoc or Mann–Whitney tests. Phe-*a* = pheophytin-a; Chl-*a* = chlorophyll-*a*; <LOQ = value lower than the limit of quantification (0.001 μg/L).

Test	NC	Cr 50 mg/L	Cr 18 mg/L	Cr 5.6 mg/L
Total cell	35,482 ± 21.17	36,893 ± 16.78	23,979	36,966
Density (cell/mL)	1419.28 ^a^ ± 14.56	1475.72 ^a^ ± 21.56	959.16 ^b^ ± 11.86	1478.64 ^a^ ± 10.76
Absorbance	0.208 ^a^ ± 0.01	0.250 ^b^ ± 0.001	0.134 ^b^ ± 0.005	0.260 ^b^ ± 0.002
Number of species	11	11	12	13
Shannon–Wiener	2.02 ^a^ ± 0.07	1.44 ^b^ ± 0.12	1.92 ^a^ ± 0.15	1.89 ^a^ ± 0.21
Evenness	0.18 ^a^ ± 0.05	0.13 ^b^ ± 0.08	0.16 ^a^ ± 0.02	0.14 ^a^ ± 0.3
Chl-*a* (μg/L)	0.012 ^a^ ± 0.002	<LOQ	<LOQ	0.012 ^a^ ± 0.08
Phe-*a* (μg/L)	0.032 ± 0.001	<LOQ	<LOQ	<LOQ
Growth rate	0.57 ^a^ ± 0.11	0.56 ^a^ ± 0.19	0.44 ^a^ ± 0.12	0.59 ^a^ ± 0.17
Growth inhibition (%)	-	−2.27 ± 1.23	22.82 ± 4.57	−2.39 ± 1.23
**Test**	**NC**	**Zn 300 mg/L**	**Zn 100 mg/L**	**Zn 10 mg/L**
Total cell	35482 ± 21.17	-	13882 ± 12.34	45504 ± 12.47
Density (cell/mL)	1419.28 ^a^ ± 14.56	-	555.28 ^b^ ± 16.54	1820.16 ^b^ ± 16.75
Absorbance	0.208 ^a^ ± 0.01	-	0.082 ^b^ ± 0.015	0.249 ^b^ ± 0.06
Number of species	11	-	9	13
Shannon–Wiener	2.02 ^a^ ± 0.07	-	1.85 ^a^ ± 0.03	1.77 ^a^ ± 0.08
Evenness	0.18 ^a^ ± 0.05	-	0.21 ^a^ ± 0.07	0.14 ^a^ ± 0.05
Chl-*a* (μg/L)	0.012 ^a^ ± 0.002	-	<LOQ	0.084 ^b^ ± 0.002
Phe-*a* (μg/L)	0.032 ^a^ ± 0.001	-	<LOQ	0.290 ^b^ ± 0.18
Growth rate	0.57 ^a^ ± 0.10	-	0.26 ^b^ ± 0.15	0.66 ^b^ ± 0.09
Growth inhibition (%)	-	-	54.65 ± 6.23	−14.49 ± 4.56
